# Perceptions about malaria among Brazilian gold miners in an Amazonian border area: perspectives for malaria elimination strategies

**DOI:** 10.1186/s12936-021-03820-0

**Published:** 2021-06-26

**Authors:** Felipe L. G. Murta, Leonardo L. G. Marques, Alicia P. C. Santos, Talita S. B. Batista, Maxwell O. Mendes, Elair D. Silva, Alexandre V. S. Neto, Marcio Fabiano, Sheila R. Rodovalho, Wuelton M. Monteiro, Marcus V. G. Lacerda

**Affiliations:** 1 Fundação de Medicina Tropical Dr. Heitor Vieira Dourado (FMT-HVD), Manaus, Brazil; 2 Universidade do Estado do Amazonas (UEA), Manaus, AM Brazil; 3 Fundação Para o Desenvolvimento Científico e Tecnológico em Saúde, Rio de Janeiro, Brazil; 4 Pan American Health Organization – PAHO, World Health Organization, Brasilia, Brazil; 5 Fundação Oswaldo Cruz, Instituto Leônidas e Maria Deane (FIOCRUZ-Amazonas), Manaus, Brazil

**Keywords:** Miners, Labor Migration, Social Perception, Malaria, Disease Erradication, Qualitative Research, Vulnerable Populations, Social stigma

## Abstract

**Background:**

Mining in the Amazon exposes gold miners to various diseases, including malaria, whose control is still a major challenge. The environment of the mines contributes to the proliferation of vector mosquitoes and the precarious housing conditions facilitate transmission of the disease. Understanding gold miners’ perceptions is essential for the formulation of strategies to fight malaria. A qualitative study was carried out in the municipality of Calçoene, state of Amapá, Brazilian Amazon adjointining the municipality of Oiapoque, that is in the border area with French Guiana and Suriname.

**Methods:**

A semi-structured interview was applied to an intentional sample of 29 miners, a number determined by the theoretical saturation criterion. Thematic analysis was adopted to obtain the results and the Cohen's Kappa index was calculated to verify the agreement between observers during coding.

**Results:**

The agreement between observers was verified by a Cohen's Kappa index of 0.82. Analysis of the interviews showed that gold miners were subjected to prejudice from the community due to forest diseases that they can transmit, and their activities are often associated with crime. When the miners return to their hometown after a period of mining, the urban population blames them for the onset of diseases such as malaria. Most participants in the survey did not know how malaria transmission occurs, and associated its occurrence with contaminated water and food. Participants reported not being afraid of the disease, trusting the diagnosis and available treatment, though this depends on where they are treated. The use of therapeutic resources, such as medicinal plants and medicines acquired in the illegal market, is very common in this population. Despite the challenges identified by the research subjects, they believe that the disease can be controlled, or the cases reduced, but there was low acceptability for a possible mass drug administration (MDA) intervention.

**Conclusion:**

Despite a recent reduction in malaria prevalence in Brazil, there are still vulnerable populations, such as gold miners, who help to perpetuate the existence of the disease in the Amazon. The lack of knowledge regarding how the transmission of malaria occurs, associated with myths regarding this and the use of traditional health practices and illegal drugs for the treatment of the disease without a specific diagnosis, jeopardizes the country’s efforts to eliminate malaria. It is necessary to implement control programmes in these populations, especially those who frequently travel around the border region and to remote locations, which are difficult regions for health teams to access, thus hindering diagnostic and treatment actions. For this reason, understanding the perceptions of these individuals as well as their customs, beliefs and lifestyle, can assist in the production of targeted educational material and adoption of strategies in the elimination of malaria in the country.

**Supplementary Information:**

The online version contains supplementary material available at 10.1186/s12936-021-03820-0.

## Background

Artisanal gold mining is responsible for the survival of many families around the world and represents a stimulus for the increasing immigration in these areas [1]. People living in gold mining regions are exposed to numerous diseases such as malaria, which in Brazil has been and still is a major challenge for the disease elimination programme [2]. Because mining work is outdoors, miners are more susceptible to mosquito bites. The dwellings in these areas, when they exist, are precarious, with many openings and are often only shacks, facilitating transmission [3–7]. The mining activity also creates a favourable environment for the reproduction of vector mosquitoes, since the dredging of ravines generates pools of water that serve as artificial breeding sites. In addition, the intense migration of workers between these areas favours the movement of infected people, increasing the possibility of transmission [6–9]. Another challenge for the control and elimination of malaria in these places is the occurrence of asymptomatic infection among the miners [10]. Several authors point to the need to consider local, cultural, social and economic aspects for the engagement of populations affected by malaria and the success of health campaigns [11, 12]. In this sense, mining in the Amazon presents itself as a challenging area for malaria control due to population mobility, cultural and social diversity and, above all, because this population is mostly located in remote and difficult to access areas [13].

In Brazil, small-scale mining, predominantly open pit mining, contributes 86% of the gold mining sector, and it is a predominantly male activity [14]. Nineteen percent of Amazonian municipalities have some form of illegal mining, located mainly in indigenous areas, environmental protection areas and extractive reserves [15, 16]. Gold mining is one of the factors associated with malaria transmission in the Americas, with various forms of exploitation. According to SIVEP-Malaria [17], malaria cases contracted as a result of activities in gold mines in Brazil contributed 3% of the country’s total malaria in 2017 and 2018 (5610 and 5427 cases respectively). The states of Pará and Amapá, due to their extensive mining activities, have the highest number of cases in mining areas.

In French Guiana, the main cause of febrile illness was *Plasmodium falciparum* malaria in individuals working in illegal mining sites [18]. The illegality of the activity constitutes a barrier to official preventive measures, and increases morbidity due to disease in the mines [19]. The health care scenario in the mining environment is complex in view of the logistical challenges for health care programmes for infectious diseases. The frequency of American cutaneous leishmaniasis in gold mining areas, especially illegal mining, is about 65 times higher when compared to areas without mining [20, 21]. Terças-Trettel et al*.* [22] reported confirmed fatal cases of hantavirus pulmonary syndrome (HPS) in gold mining regions in the Amazon. Considering that hantavirus and malaria have a similar clinical manifestation in the prodromal phase and that they occur concurrently in the studied gold mining region, the authors suggest implementing differential diagnosis for these patients, as the high frequency of malaria, chikungunya, dengue, zika, leptospirosis and others in mining populations were determinant in the fatal evolution of the cases reported [22].

Mining activity in the Western Brazilian Amazon has increased in recent years driven by the opening of new mining areas after the saturation of old areas such as Serra Pelada and Tapajós in Pará [23]. From a historical social perspective, artisanal miners were important for the exploration and settlement of the Amazon region, especially in the 1980s. However, their constant mobility, associated with violence and illegal practices such as deforestation and forest pollution, make this population marginalized and, as a result, the prospectors are seen as disqualified, adventurous and stigmatized [24]. Mining in the Amazon is generally a structured activity, with well-defined work functions in which manual workers, entrepreneurs and traders participate. It primes the economy of nearby cities and attracts immigrants. Many land conflicts and accidents occur in these areas, especially in new mining areas. Therefore, small-scale mining activity is considered a high-risk job [25].

Despite decreased malaria prevalence due to improvements in diagnosis, treatment and awareness campaigns, there are still several barriers that need to be overcome in order to achieve its elimination in Brazil. Along with indigenous groups and the rural population, gold miners have a role in maintaining the disease in the Amazon, as they often travel across the border region between Brazil, Guyana, French Guiana and Suriname in order to establish themselves illegally in remote mining sites, making it difficult for health teams to carry out surveillance, diagnosis and treatment actions [8, 26]. Thus, often this population ends up being unassisted in these areas of difficult access and may self-medicate inappropriately, increasing the risk of selection of resistant strains of *P. falciparum*. Nacher et al*.* [27] drew attention to the fragility of surveillance of this population and highlight a possible emergence of *P. falciparum* resistance to Coartem® in this region, a factor that undermines cross-border efforts to eliminate malaria in the Americas [27–30].

Mass drug administration (MDA) to the entire population is used to prevent, control, or eliminate diseases like malaria, whereby antimalarials are administered periodically, even if the participants do not have the symptoms of the disease in question [31, 32]. A recent study that used the spatially explicit model of malaria transmission showed that an MDA strategy in *P. falciparum* malaria is suitable for mobile populations in an elimination context [33]. However, MDA strategies must consider the acceptability of the target population. There are several challenges for the development of health education projects with mobile population such as low schooling, ignorance of the health information of the different places where they pass and the logistics of access to places of residence and work [34]. These projects must essentially consider language and forms of communication that best suit this population [35].

Understanding how miners perceive malaria and what they think about the tools used in surveillance, control and disease elimination programmes is essential for the development of communication strategies and educational materials for this population. The objective of this research was to characterize the perception of malaria among Brazilian gold miners and the acceptability of disease control measures already used in gold mining areas and evaluate the acceptability of the idea of an MDA intervention.

## Methods

### Study area and recruitment of participants

**Fig. 1:**
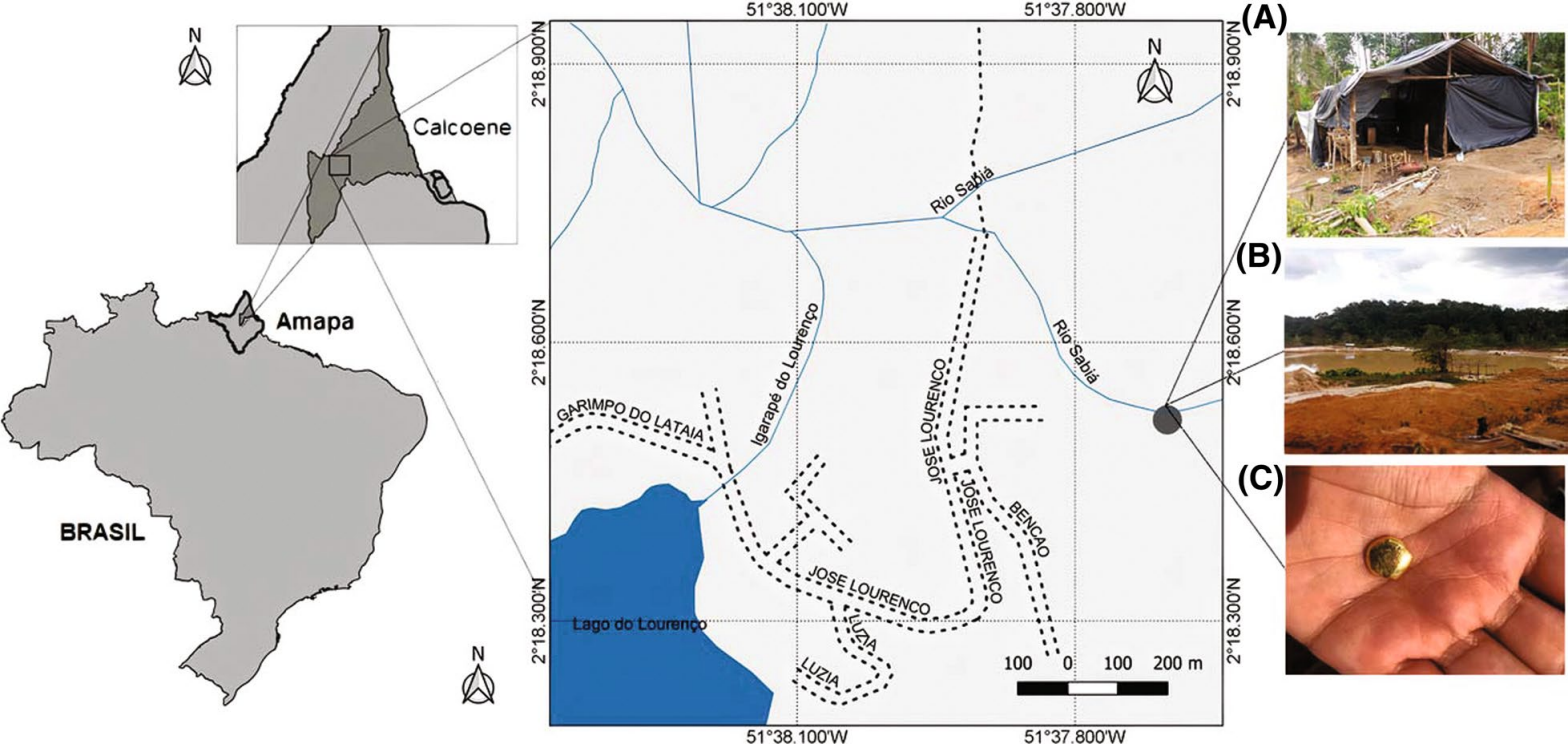
Study area. **a** camp where the miners sleep; **b** Artificial lake formed due to mining activity; **c** Gold mined in the region after 1 month of work by a gold miner interviewed by us

The study took place in a mining region called Lourenço, in the municipality of Calçoene, state of Amapá, near the Brazilian border with French Guiana. Gold mining areas are rudimentary and offer little infrastructure for the miner (Fig. 1).

**Fig. 2:**
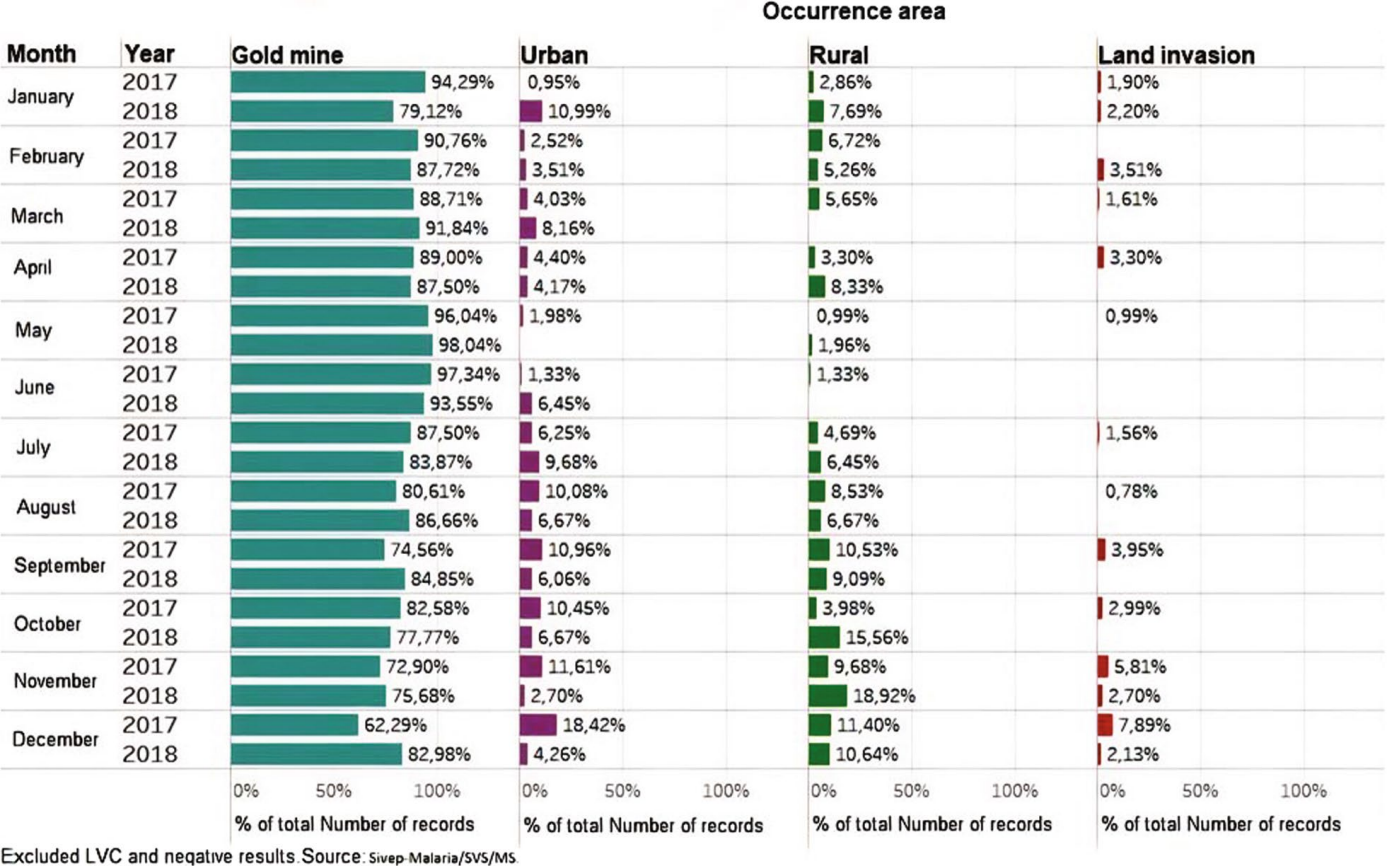
Occurrence of malaria cases in mining, rural, urban and settlement areas in the municipality of Calçoene, Amapá, Brazil

The last census (2010) estimated the local population of Calçoene at 1866 inhabitants, with about 50% living on 500 Brazilian Reais a month (equivalent to $92-08/11/2020) [36]. According to the latest Brazilian Institute for Geography and Statistics (IBGE) survey, in 2010 the municipality of Calçoene ranked an average of 0.643 in the Human Development Index, with an estimated population of 11,306 people in a territorial area of 14,117,297 km^2^, which represents a demographic density of 0.63 inhab/km^2^. In 2017, Calçoene presented the highest incidence of malaria in the mining area in the state of Amapá, accounting for 83% of cases. That year, 1248 cases of malaria were registered in the city. In 2018, there was a significant reduction in the absolute number of cases, however gold mining areas continued to be responsible for the highest malaria incidence rates in the region (Fig. 2) [17].

Lourenço is an old mining area whose main phase of exploration took place in the 1990s, by a large mining company that explored the gold deposits until 1995, when the commercial extraction of this ore was no longer profitable, taking away all the machinery [9, 37]. Artisanal miners then began to explore the area and created a cooperative that operates in the region, with less elaborate equipment and inferior extraction capacity [38]. In 2018 there was 31 active mining sites around Lourenço, the distance between the closest mining area from Lourenço is 1 km, and the furthest is 20 km away and frequently new mining sites have been discovered, thus this distance variation there is a constant mobility from these workers [17].

There is only one basic health unit in Lourenço responsible for primary care, such as diagnosis and treatment for leishmaniasis, Chagas disease and malaria. The health unit has 3 endemic diseases agents responsible for the active search and treatment of malaria cases, in addition to 2 microscopists. The principal method used to diagnostic malaria in the region is thick blood smear collected during the active search and passive case detection. Rapid diagnostic tests are used during two specific situations: a) when electric power goes off, which occurs frequently in the region; b) when the endemic agents go to the furthest areas in the mining sites, where is necessary to perform the diagnostic during the field. In 2019 of the 573 cases notified in the region, *Plasmodium vivax* was responsible for 569, while *P. falciparum* registered only 4 [17]. In Brazil, the diagnosis and treatment of malaria and other infectious diseases are made free-of-charge by the Brazilian Unified Health System (SUS). In cases of *P. vivax* malaria, a combination of chloroquine and primaquine is used, according to the patient's weight, with the treatment lasting up to 14 days. For cases of *P. falciparum,* a combination of artemether/lumefantrine is used [39].

### Study design

After contacting the local leader of the miners, who facilitated interaction with the gold miners face-to-face, the researchers explained to the miners about the study, and those who agreed to participate were recruited, then an in-depth interview (IDI) was conducted with an intentional sample of 29 miners. This number was determined by the principle of theoretical saturation where IDIs are carried out until a clear pattern appears and subsequent groups do not produce new information [40, 41]. Two workers declined to participate, claiming that they did not feel comfortable with the questions since many engage in illegal mining activities in the region. A semi-structured interview guide was used with 22 open questions, complementary questions and instructions that allowed the interviewer to investigate a topic in more detail (Additional file 1). The questions were developed by Qualitative Research Team (QRT), an experienced research team that works with malaria and addressed questions regarding elimination and major malaria control strategies, based on the study by Murta et al*.* [42]. The interview guides were previously tested and validated by the researchers in a smaller sample of volunteers and adjustments were made to ensure the transparency and relevance of the questions.

The IDIs were carried out in a basic health unit in the city, in a comfortable and quiet room where the interviewer, the observer and the participant were accommodated. The interviews lasted an average of 40 min and the field notes were recorded by the interviewer and also by the observer. The interviews were recorded, transcribed and inserted in the MAXQDA 20 programme, without personal identifiers, so that the database could be anonymized.

The qualitative analysis was carried out through a thematic framework, with the elaboration of categories that emerged during the analysis process (inductive and deductive coding) after the previous reading of the transcripts. These categories were discussed among three researchers in order to build consensus. In addition, two researchers independently developed a codebook and performed line-by-line coding.

The data collected were triangulated as a strategy for validation of the results and a more reflective analysis of the data. Although the team had experience with qualitative research and malaria, they had not previously interacted with the gold mining population, which stimulated the process of reflection on the data collected. Cohen’s Kappa index was calculated in order to verify inter-observer agreement after a first coding; we obtained a Cohen’s Kappa score of 0.53 showing moderate agreement. After a new discussion regarding the analytical categories, we obtained a Cohen Kappa score of 0.82.

While developing the study design of this manuscript, the consolidated criteria for reporting qualitative research (COREQ) were followed: a checklist with 32 qualitative research items in order to ensure that a high-quality report is obtained (Additional file 2). COREQ items help researchers to report important aspects of the research team, the methods used in the study, the context of the study, the analyses, and the interpretations of the results.

The research team consisted of eleven members (FLGM, LLGM, APCS, TSBB, MOM, EDS, AVSN, MF, SRR, WMM, MVGL). All team members are specialized professionals working in the health area and have experience in qualitative research and publishing. The study coordinator contacted the participants and, to avoid any undue influence, another team member conducted the interview. The team members made every effort to avoid the influence of personal views and biases during data collection and data analysis, and to prevent bias during the writing stage of the study.

## Results

### Social characteristics of participants

**Fig. 3:**
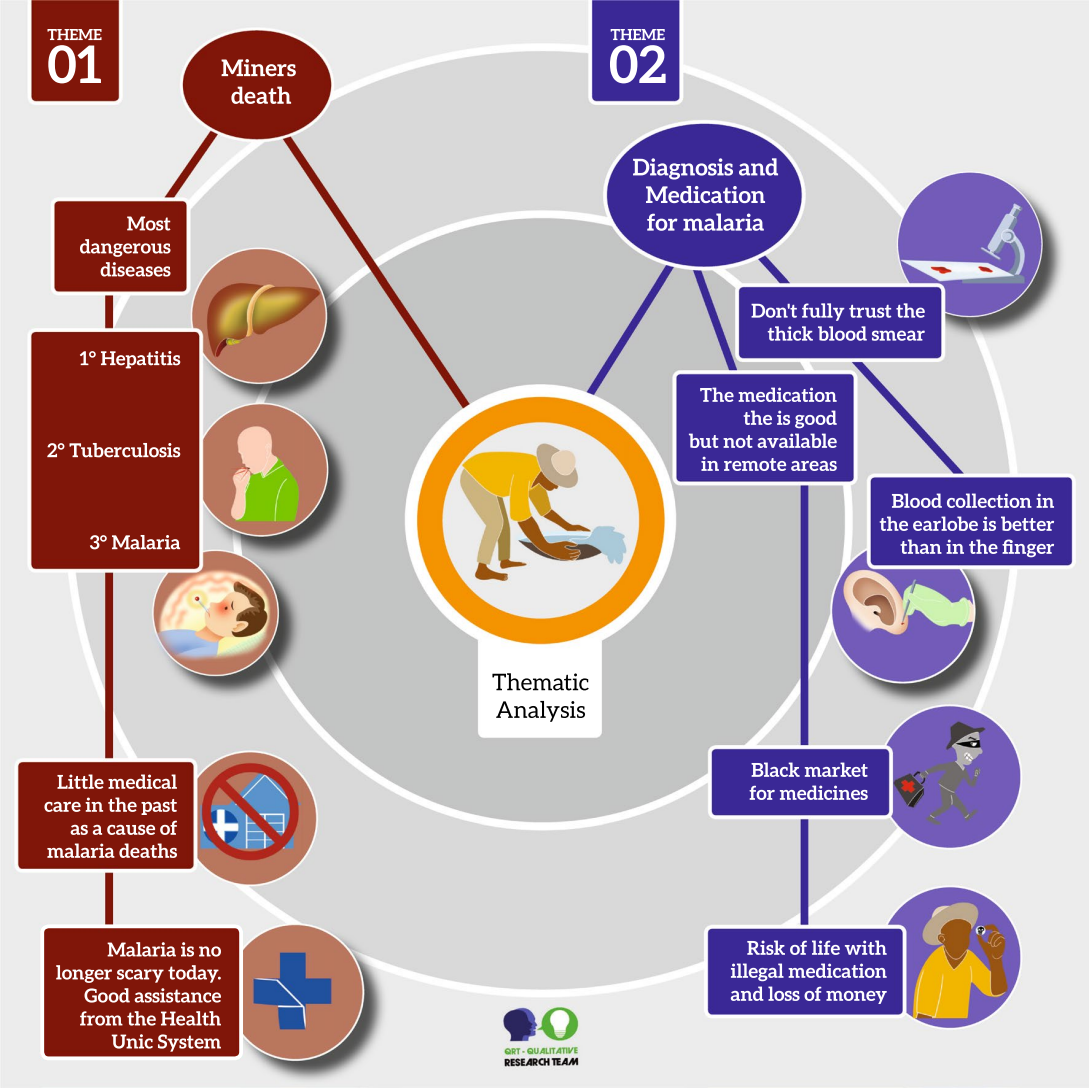
Representative scheme with the main results of the thematic analysis of the interviews

**Fig. 4:**
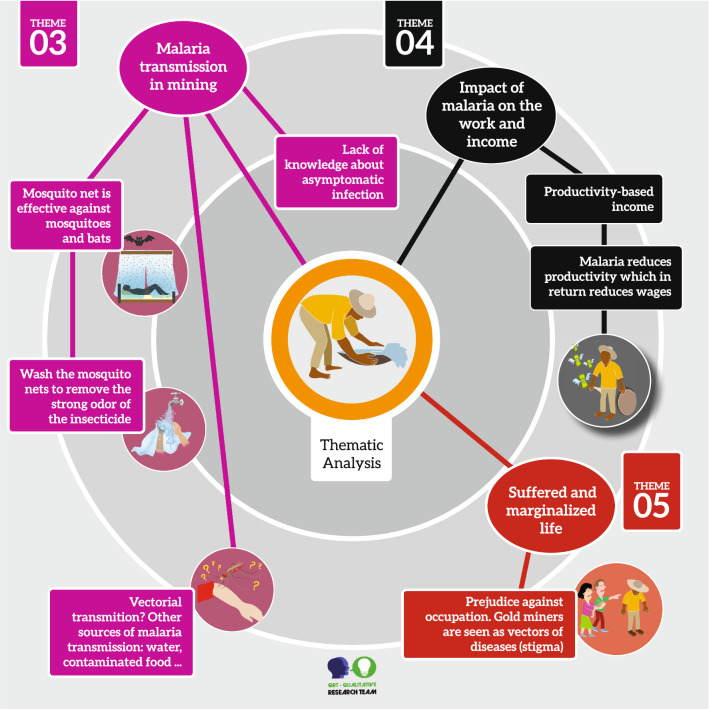
Representative scheme with the results of the theme 03, 04 and 05

Mining is an activity predominantly performed by men. Only one person interviewed was female, who worked as a cook in the mining operation. The average age of the participants was old 47 ± 10 years and the predominant level of education was elementary school, which all the interviewees had completed. Five participants were illiterate and the average length of service in the mine was 20 (± 6) years. The analysis of the 29 interviews and the field notes allowed us to identify the five major themes, which are illustrated in Figs. 3 and 4.

### Theme 1—Death in mining

Most of the participants have already witnessed death from diseases in the mine. Hepatitis, tuberculosis, and malaria were cited as the main cause of death from infectious diseases. Regarding malaria, 16 participants (55%) reported cases of co-workers who had died from the disease. Most died due to lack of medical care or incorrect treatment. However, most reports were from participants who experienced part of the gold rush in the 1970 and 1980s; a time when there was not much infrastructure in the region or the Brazilian National Health Service (SUS), as well as decentralization in the diagnosis and treatment of malaria in the Amazon Region.

“One memory that struck me was seeing my friend die of malaria, where he was not diagnosed and had the wrong treatment, because we thought he was just suffering from a virus, and when he died, the autopsy found that it was malaria, and he died without receiving help and this is very sad” (Gold miner 5).

“...I only remember those in which the miners don’t take all the doses and end up drinking alcohol and sometimes there is no way to cure them when they go seek treatment. They are already very bad and end up dying. They even made a cemetery for the miners. And do you know what happens there? Some people dig up the bodies because some were buried with many gold nuggets” (Gold miner 19).

“When I came here to cook, there were no women working in the mine because the work was heavy and there was a lot of malaria, I was at the brink of death because of malaria” (Gold miner 27).

Regarding the current malaria situation, the miners reported that they have lost their fear of the disease, despite becoming infected, since they know that if they get sick, they will have access to diagnosis, doctors and medicines.

“Malaria no longer frightens anyone, nowadays it is no longer frightening, because there is medicine available for everyone and this makes life much easier because if the gold miner gets sick he can go to SUCAM (Superintendence of Public Health Campaigns) and gets medicine” (Gold miner 19).

“In the past I was afraid to go to the mine because of malaria, and I heard stories and saw people with symptoms and that made me afraid, nowadays I am no longer afraid of malaria” (Gold miner 3).

### Theme 2. Diagnosis and treatment of malaria

Regarding the thick smear test, despite the great majority expressing confidence in the test, some interviewees reported not fully trusting the test. These interviewees reported that the finger was not the best place to collect blood as it would run the risk of a false negative result. They claimed that they prefer blood collection in the ear lobe because they reported the occurrence of false-negative results in a sample collected from the finger and positive results for collection in the ear lobe.

“...sometimes it doesn’t give a positive result either on the finger exam or on the vein exam, but also if they collect blood from the ear that gives a positive result” (Gold miner 9)

The interviews revealed that workers believe in medicinal plants. Bottles containing infusions of roots and leaves of common plants of the Amazon are very common among miners. They believe that quinine tea, bilberry tea, paca gallbladder tea (*Cuniculus paca*, a wild rodent that is common in the region) possess medicinal properties. Despite this, some said they did not agree with these practices for treating malaria due to negative experiences in the use of these popular and home remedies.

“I have already heard of boldo* tea for malaria and several other teas made from tree bark. I have already had some, and people say that they have taken them and have been without malaria for over twenty years. These bottles of infusions are very good” (Gold miner 22).

*Peumus boldus.

“I've heard of malaria teas and bottled infusions, one of which is gall tea, but I never got to take these homemade medicines. But I've heard reports from people who took them and never had malaria” (Gold miner 15).

“Some gold miners I knew died because of that, they stopped taking the normal medicine to take the natural ones and ended up not having the desired effect against malaria and they died” (Gold miner 25).

Most participants trust the medicine distributed by the National Health Service in Brazil. However, many respondents reported discomfort with the medication due to the adverse effects of the treatment. Many complained of having to travel from the mine to the city for a diagnosis and for medicines. This displacement, according to the miners, could be avoided if they had the medicine available in the mining area.

“Some people are able to take malaria drugs to the mines, because they ask the doctor and take it in case they start to feel any symptoms. Then, take it to the mine without needing to come here again” (Gold miner 5).

Several gold miners reported resorting to the clandestine antimalarial market when they are in the mines and start to show symptoms of the disease. Reports indicate that Artecom® (dihydroartemisinin-piperaquine), which is unregistered in Brazil, illegally enters the country through the borders with Suriname, Guyana and French Guiana. The miners describe that the drug has the advantage of being a single dose, which relieves symptoms for a few days, but does not cure the disease. The choice of medication by the miner depends on where he is when he becomes symptomatic. Respondents reported that geographical isolation, in addition to the loss of work days if they have to return from a distant area to the city, are major factors in choosing this treatment, regardless of knowing the type of malaria. The miners interviewed were unaware of the importance of different treatments for different types of malaria.

It should be noted that the cost of Artecom® in the mines, according to respondents can be as much as 300 Reais ($55 –08/11/2020), with a significant economic impact, representing approximately 60% of the miners' average monthly income as well as their health.

“...people take single dose medicines without even knowing what type of malaria they have, without having an exam, without anything.” (Gold miner 11).

“I've already taken Artecom, but I think that these remedies* here in Brazil are even better than what Artecom is. Even some miners prefer these remedies here in Brazil, despite being a bad medicine, but they provide security for the patient. With these drugs, the patient takes a complete treatment.” (Gold miner 22).

*The respondent refers to chloroquine and primaquine or Coartem® distributed free in Brazil upon specific diagnosis of the disease.

“But there is a medicine that is a single dose that comes from another country. It was strong, but the good thing is that it was a single dose.” (Gold miner 18).

Respondents reported that the number of pills and the length of the treatment were two disadvantages of the medication distributed by the government in Brazil. Treatment abandonment, mentioned by the miners, is very frequent, caused mainly by the ingestion of alcoholic drinks. Alcoholism in mining was mentioned as a common problem, associated with a hard-working life and isolation. Many cited the use of brandy as the main drink due to its affordable price and the lack of need for refrigeration.

“Here many people abandon treatment because of alcoholic drinks. Because people take a few doses of the medicine and they already feel well and this causes the miners to abandon the treatment because they are feeling good already, and they will start to consume alcohol again” (Gold miner 1).

“Practically every month I had malaria, but this was due to my carelessness. Even though I had the disease, I only took the medicine until Friday, because on Saturday I stopped taking it to drink alcohol” (Gold miner 17).

Some miners perceived the medication as a contributing factor to the death of some patients. According to them, depending on the person’s clinical condition, their bodies could not handle the treatment.

“I’ve seen people die of malaria. They arrive very weak because the disease is very strong and when they took the dose of the medicine, which is very strong, they ended up not being able to handle it and that was fatal.” (Gold miner 21).

“The medicine is very strong, you cure malaria and get sick in the liver due to the medicine and this is very bad...” (Gold miner 03).

MDA is a tool used in several countries as a strategy to combat malaria. During the interviews, the researchers asked about a possible MDA intervention in the mine. The miners showed resistance to the implementation of a programme like this and, with the main refusal reason being a need to be symptomatic to take the medication. According to them it is necessary to perform the malaria test. In addition, they reported the negligence of the miner regarding his own health as an important factor for refusing an MDA programme.

“They wouldn’t take the medicine if they didn’t feel anything, the miner is stubborn and without feeling anything he wouldn’t consider himself sick” (Gold miner 5).

“I think it would not make sense to give medication without the person feeling anything wrong with him, and without having a positive test. If I tested positive for malaria, and I don’t have symptoms I wouldn’t take anything, because this is a cultural thing for the miner, he only takes medicine when he feels the symptoms and it’s already bad” (Gold miner 4).

“Here in the gold mine, it is difficult for people to accept mass medication, because here people only take it when they are really feeling the disease, without an exam they wouldn’t take it” (Gold miner 1).

### Theme 3. Malaria transmission and prevention in mining

The transmission of malaria by mosquito bites was reported by most respondents. However, the statements given on this topic evidenced doubts, and were always accompanied by "I think", "because people say". As such, they seem to be based on popular beliefs.

“Some say you get malaria from the mosquito, but I'm not sure about that, because it can also be caught from water or some other virus” (Gold miner 13).

Doubts about the transmission of the disease led to many theories regarding the source of infection. People believe that they could be infected through contaminated water, viruses, environments such as ponds, and poor diet.

“You get malaria by drinking dirty water, water from rivers, the malaria I got was like that, when I was in the forest drinking water directly from rivers.” (Gold miner 5)

Among the 29 workers interviewed, only one participant showed confidence in his statement regarding how malaria transmission occurs. About half of the interviewees did not believe that people could be asymptomatic for malaria and reported that cases of malaria increased during the rainy season.

“I think you only have malaria if you have symptoms, otherwise there is no way you have malaria” (Gold miner 2).

“Malaria in the rainy season here is cruel, we already had a record level of malaria in the municipality and, in the neighboring community, all the residents had malaria.” (Gold miner 29)

The insecticide-impregnated mosquito net was cited by most miners as an effective measure and used for fear of malaria or other animals such as bats, since the mosquito net becomes a physical barrier during the worker’s sleep. The washing of the mosquito net in order to remove the strong odor it exhales was reported by the interviewees.

“There are people who are not used to using it and are very uncomfortable with the use of mosquito nets. Now those who have had a lot of malaria like me use the mosquito net out of fear.” (Gold miner 7).

One of the reasons reported by the participants for not using the mosquito net was the distance between the city and the mining area, because according to them, if the distance is short, there is no need to sleep in the forest. In addition, in the city, due to the availability of electricity, they can use a fan, as they believe that this measure keeps mosquitoes away.

“I don’t use the mosquito net much because I don’t sleep in the woods” (Gold miner 11).

“I don’t use it so much because I use the fan and the fan on its own is enough to keep the mosquito away” (Gold miner 15).

### Theme 4. Impact of malaria on work and income

The biggest problem with infectious diseases such as cutaneous leishmaniasis and malaria in mining is that they limit the productivity of the worker. Some type of economic loss caused by the disease was cited by all respondents. Respondents reported having a fragile and informal employment relationship, where a worker can be replaced by another due to their health status. Most prefer to return to work as soon as the main symptoms disappear, around the third day of treatment.

“...because if he gets sick and doesn’t work, he doesn’t get paid and I think that this is also a factor for treatment abandonment, with three days of treatment the person already has to go back to work, where many times they will have to fight for a place with the person that has already replaced him. This return to mining after getting sick is very complicated, because if he cannot return, he will have to seek other camps to be able to work. This has happened to me a lot.” (Gold miner 6).

“I have seen people missing work due to malaria, but I still haven't had malaria and, if I did get it, I would wait between three and four days and then go back to the job” (Gold miner 23).

“When I got malaria, I had to leave the team of miners I was part of, sometimes they even accept you back when you get better, but most often have to look for another team to work with.” (Gold miner 7).

“...when you work for the machinery owners and the miner gets sick with malaria, the owner hires someone else to work because the miner has no support from anyone” (Gold miner 29).

### Theme 5. Prejudice and marginalized life

The miners reported that their job was not a life choice, but a life chosen for them, due to the few opportunities they had in childhood. Most of them started mining as a child or teenager and reported luck and hope as the main factors that motivate them to take risks every day in search of gold. Interviewees reported that they do not feel welcomed by society because of the work they do, since there is an association between crime and mining, a fact that, according to them, has no basis in reality. They also cite the environmental conflict they experience as a result of mining activity that, according to the interviewees, lacks efforts from authorities to teach the correct forms of mine management in order to minimize the environmental impact.

“Mining is luck, I was lucky once in my life, but I think about having another source of income. Here is what we have at the moment as a job, because it was where we grew up doing it and we ended up enjoying it, but if I had the opportunity to change I would change.” (Gold miner 8).

“The life of the miner is very hard, and society sees us as invisible people, as outcasts and bad people, and there is a lot of prejudice against people who work in mining. Gold mining is about luck. It’s hard to get a spot, and it’s much harder to find the gold.” (Gold miner 9).

“Society sees us with evil eyes, especially the environmentalists who say that we only degrade the environment. They just point fingers and come to inspect, but no one comes to teach us how to do it correctly.” (Gold miner 20)

“People think the miners are wild, that they end up with everything they go through, but it just isn’t like that. There are a lot of good people here, and we are only fighting for survival because the government does not provide good working conditions.” (Gold miner 27)

Interviewees also reported prejudice regarding the health status of the miners. Since there is a higher incidence of some diseases among miners, when these workers return to the cities, they end up being victims of this stigma.

“They end up suffering a certain prejudice, especially when it comes to malaria. This is because when we go to the city they say that if malaria infects many people it is the fault of the miner who brought malaria from the mine.” (Gold miner 16).

In spite of the challenging experiences of the miners such as death caused by the disease, poverty, prejudice, poor living conditions and addictions, such as alcoholism, workers showed optimism regarding the reduction of malaria cases and they even believe in elimination. However, they stated that it would require a large investment from the government, commitment and scientific research, in addition to the collaboration and participation of the population affected by malaria.

“I think there is a way to end malaria in Brazil, but it would be a lot of work and, in order to end, the work would have to be continuous, and more constant, to use this smoke with poison more often, not only where people live, but also where they don’t live” (Gold miner 10).

“It is very complicated to put an end to malaria, but it can be reduced, because there is no way to eliminate all the mosquitoes from this huge forest. But that the government can intervene and invest well in the whole of Brazil to end malaria.” (Gold miner 18).

“The Brazilian government has to set a goal. It needs to be more committed to the people, to know the geography of the locations affected by malaria and to invest money so that it is eradicated.” (Gold miner 27).

## Discussion

Brazil, a country with a large mineral reserve in its subsoil, often experiences gold rushes, especially in the Amazon region, which still has unexplored deposits [43]. Infectious diseases, such as malaria are part of daily life in mines in the Amazon, and negatively impact the lives of workers and their families since this population is mobile and is constantly migrating. The working population of the studied mine was mostly composed of workers with low education levels, being 20 years the average length of service in the mine. Reports of death from malaria in mines were mainly associated with a time when there was no National Health Service in Brazil and malaria was the main disease that affected miners. Currently, it is clear that the disease is no longer the main cause of concern for these workers, due to the recent reduction in malaria associated mortality in Brazil [42].

The drugs for treating malaria in Brazil are free but require prior diagnosis at a health facility. One of the complaints made by the miners, especially those who were diagnosed with *P. vivax* malaria, is the amount of pills to be taken over a period of up to 14 days, which make it difficult to return to work. Perhaps it is important to think of miners as a target group for the use of medications such as tafenoquine instead of primaquine, since tafenoquine can provide radical cure of *P. vivax* malaria in a single dose, which could potentially result in greater adherence to treatment [44]. However, tafenoquine requires a diagnosis of the G6PD status of each individual, since it can cause acute haemolytic anaemia as seen in a study in the western coastal region of Southwest Sumba [45]. Brito-Sousa [46] recently reported the challenges during a mass G6PD screening in the Brazilian amazon, it showed good acceptability by the population, making it viable to perform mass G6PD testing in endemic malaria regions in Brazil. The consumption of alcoholic drinks was cited as a factor for low adherence to *P. vivax* malaria treatment. Some authors point out that excessive alcohol consumption is one of the main health problems in the mines and is a factor that, associated with others such as mercury contamination, reduces the life expectancy of this population [47]. In Brazil, in Serra Pelada, a region of intense mining in the 1980s, there was a myth among workers that those who did not spend their money on women and drinks would not find gold, which contributed to the increase in cases of alcoholism and consequently cirrhosis among the miners in the region [43].

Traditional knowledge and the use of medicinal plants to cure physical diseases has been reported throughout the history of the riverine population of the Amazon. This custom is strongly rooted in the culture and is due mainly to the influence of the indigenous people in the region that needed to develop survival strategies in the jungle using the natural resources that were available. The places where many riverine dwellers live are difficult to access, far from urban centres, but still need medical assistance. The use of traditional medicine is common in the Brazilian Amazon, even in populous cities in the state of Amazonas, where it was observed that the population cultivates and collects medicinal plants in their backyards [48].

Many people in the Amazon region use parts of animals, including the lard or oils obtained from them, to treat diseases. The list of species used is extensive, and includes alligators, snakes, jaguars, various fish and terrestrial mammals, including the peccary, a species of wild pig (*Tayassu pecari*) that is used by some riverside dwellers to treat malaria [48, 49].

The consumption of anti-malarials that are illegal and of unknown origin was pointed out as a solution for miners who are unable to obtain medication legally due to the distance between the nearest health unit and the ore extraction area. This self-medication, carried out without guidance and without a specific diagnosis, can hinder government efforts to eliminate malaria, especially in Brazil [8]. One of the suggestions pointed out by the interviewees was to make medicines available in such a way that the miner could transport them to these remote areas and perform self-medication when he perceives the first symptoms. Douine et al*.* [50] tested this self-diagnosis and treatment approach in illegal mining areas on the border between Brazil and French Guiana. The researchers provided kits for rapid diagnosis and the respective medications to miners who transit through this region and have since monitored participants to assess improvement in treatment in this population and evaluate whether transmission is reduced. The approach “If the mountain won't come to Muhammad, then Muhammad must go to the mountain” may be the key to success in strengthening malaria elimination programmes in countries that have a high incidence of malaria in this specific population because it empowers that population over their health situation. Another important aspect that this approach makes possible is their monitoring, since they need to collect the drugs and kits and, in doing so, they can provide important data on the epidemiology of the disease, especially when signs of decreased susceptibility to artemisinin have been observed in some isolates of *P. falciparum* in Suriname [51–53].

Regarding knowledge about the transmission of the disease, there was a lack of knowledge about vector transmission. Many still associate the occurrence of malaria with drinking contaminated water, as there are rarely safe sources of drinking water in these workplaces. Studies in other countries have shown that people affected by the disease strongly believe that there is a relationship between malaria and food [42, 54, 55]. Education and permanent communication in health is essential so that scientific knowledge can be incorporated by the population, thus helping in preventive measures [56].

Adhesion to the use of mosquito nets impregnated with insecticide (long-lasting insecticidal nets) is considered one of the main vector control measures for reducing the incidence of malaria. Discomfort with heat and skin irritation are negative factors associated with use of the mosquito net, as reported in other studies [42, 57]. However, when the miners are in the city, the fact that they believe that by using a ventilator fan they will be protected against the disease must be considered and, as such, addressed in health education campaigns.

The working relationship that exists in the mines, in which the worker obtains his income according to his productivity, makes infectious diseases, such as malaria, a reason for intense income loss, leaving this population in a greater situation of social vulnerability. Therefore, due to this informality, this worker lacks access to the social security system and starts to rely only on his financial reserves in case of illness. There is also an impact on treatment since the labour relationship in the mine forces the worker to return to his activities before even finishing the treatment. This can lead to abandonment of primaquine and maintaining hypnozoites in this population, making it difficult to achieve malaria elimination in Brazil.

## Conclusion

Brazil has reduced the incidence of malaria in recent years in a continuous effort that has proven effective. However, there are still several challenges to be overcome in the country in order to achieve elimination of the disease, and asymptomatic malaria infections among miners is perhaps one of the greatest. There are strategic malaria control programmes in Brazil, however, they are not as effective in gold mining areas mainly due to the logistics of access to this population and the illegal aspect of some mining areas. The gold miners’ perceptions are fundamental for the construction and implementation of appropriate control and treatment measures for this specific population. Thus, when we know how this population perceives the disease, and understand their customs and lifestyles, we can design educational strategies to improve adherence to existing programmes or even reformulate access strategies for this population. Another important aspect to be considered and raised in the study is the impact of malaria on the productivity and income of these miners and the adoption of new tools, such as tafenoquine, which can contribute to reducing treatment time and increasing compliance. The stigmatization that the population suffers must also be considered in control programmes and new educational materials, so that this problem is avoided. The materials destined for gold mining areas must contain accessible language and predominantly use visual resources, such as images, since the education level of this population is low, which could compromise health communication.

## Supplementary Information

**Additional file 1.** In-depth interview guide. http://dx.doi.org/10.17632/dfjsc94s7c.1

**Additional file 2.** Consolidated criteria for reporting qualitative research (COREQ). https://doi.org/10.6084/m9.figshare.14342675.v2

## Data Availability

The data that support the findings of this study are available at Fundação de Medicina Tropical Dr. Heitor Vieira Dourado. However, restrictions apply to the availability of these data, which were used under license for the current.

## Abbreviations

COREQ: COnsolidated criteria for REporting Qualitative research
G6PDd: Glucose-6-Phosphate Dehydrogenase Deficiency
HPS: Hantavirus Pulmonary Syndrome
IBGE: Brazilian Institute for Geography and Statistics
IDIs: In-Depth Interview
MDA: Mass Drug Administration
SUCAM: Superintendence of Public Health Campaigns
SUS: Brazilian National Health Service
