# Supplementary material for: Perceptions about malaria among Brazilian gold miners in an Amazonian border area: perspectives for malaria elimination strategies
Source: Malar J. 2021 Jun 26;20:286. doi: 10.1186/s12936-021-03820-0 (PMC8236171; doi:10.1186/s12936-021-03820-0)
Supplement: Supplementary file 1 — Additional file 1. In-depth interview guide. http://dx.doi.org/10.17632/dfjsc94s7c.1 [file 12936_2021_3820_MOESM1_ESM.pdf]

FUNDAÇÃO DE MEDICINA TROPICAL DOUTOR HEITOR VIEIRA DOURADO
INSTITUTO DE PESQUISA CLÍNICA CARLOS BORBOREMA

TEAM:
ALÍCIA PATRINE
LEONARDO LINCOLN
MAXWELL MENDES
TALITA BASTOS

COORDINATOR: Dr. FELIPE MURTA

SEMI-STRUCTURED INTERVIEWS GUIDE TO EVALUATE THE PERCEPTIONS ABOUT MALARIA AMONG BRAZILIAN GOLD MINERS IN AN AMAZON BORDER AREA: PERSPECTIVES FOR MALARIA ELIMINATION STRATEGIES.

MANAUS – AM
2021

SEMI-STRUCTURED INTERVIEW

1. **Thematic axis: Current malaria scenario and control measures.**

- How do you see the current situation of malaria in the mines? (increasing, decreasing)
- What is the impact of malaria on the mining you work at?
- How do you think malaria is transmitted?
- During what time of the day do you think people get malaria? (morning, afternoon or night)? Why?
- Have you ever had malaria? How was it?
- Which malaria control measures do you think are most effective?
- What do you think of *Fumacê*? (Strategy where a "cloud" of smoke is used with low doses of a pesticide that eliminates most of the adult mosquitoes present in the region)
- What do you think of home spraying with pesticide?
- What do you think about the use of Long-lasting insecticide-treated nets (LLINs)?

2. **Thematic axis: Gold mining and diseases.**
    - Have you heard of other diseases that people caught in mining? If so, could you name them?
    - Which disease are you most afraid of getting in the mines? Why?

3. **Thematic axis: Life stories.**

a) Do you have any memories/stories about malaria?

- If so, who was associated with the story?
- Was it someone from your family?
- Where did it happen? When was it?
- How does the story end?

4. **Thematic axis: Diagnosis and treatment.**

- Do you think the malaria test is reliable? Why?

- Do you think it is possible for a person to have malaria and not feel anything? No symptoms?
- What do you think of the malaria medication? How to get it in the mines?
- Have you ever heard of bottles of malaria medication or teas to treat malaria?

5. Thematic axis: Elimination and mass treatment.

- What do you think about treating all people in a neighborhood/city who have cases of malaria with medication and without prior diagnostic tests? The treatment will be done as follows: everyone will take the medication; people who have symptoms for malaria will take the medicine and people who have no symptoms for the disease will also take the medicine. This will be done to treat people who have the malaria parasite, but who do not experience any symptoms and do not know they have the disease.

- Do you think we could end malaria in Brazil? How could this be done? What the government could do?

- What could be done in mining to improve the malaria situation?

These were all of our questions, if you have any questions I would be happy to answer you. Thank you very much for your participation in this interview.
