# Supplementary material for: Perceptions about malaria among Brazilian gold miners in an Amazonian border area: perspectives for malaria elimination strategies
Source: Malar J. 2021 Jun 26;20:286. doi: 10.1186/s12936-021-03820-0 (PMC8236171; doi:10.1186/s12936-021-03820-0)
Supplement: Supplementary file 2 — Additional file 2. Consolidated criteria for reporting qualitative research (COREQ). https://doi.org/10.6084/m9.figshare.14342675.v2 [file 12936_2021_3820_MOESM2_ESM.pdf]

This website uses cookies to help you have a better on-line experience. By using this website, you are agreeing to the use of cookies as explained in our cookie policy.

Accept cookies

figshare Log in Sign up

Consolidated criteria for reporting [illegible]
32-item checklist

Developed from:
Tong A, Sainsbury P, Craig J. Consolidated criteria f[illegible]
32-item checklist for interviews and focus groups. *Int*[illegible]
2007. Volume 19, Number 6: pp. 349 – 357

No. Item	Guide question[illegible]

COREQ_Garimpo.pdf (162.17 kB)

COREQ_Garimpo.pdf

About Features Tools
Blog Ambassadors Contact
FAQs Privacy Policy Cookie Policy
T&Cs
Sitemap
